# Supplementary figures and images for: Dental caries as a risk factor for bacterial blood stream infection (BSI) in children undergoing hematopoietic cell transplantation (HCT)
Source: PeerJ. 2022 Sep 23;10:e14040. doi: 10.7717/peerj.14040 (PMC9511999; doi:10.7717/peerj.14040)

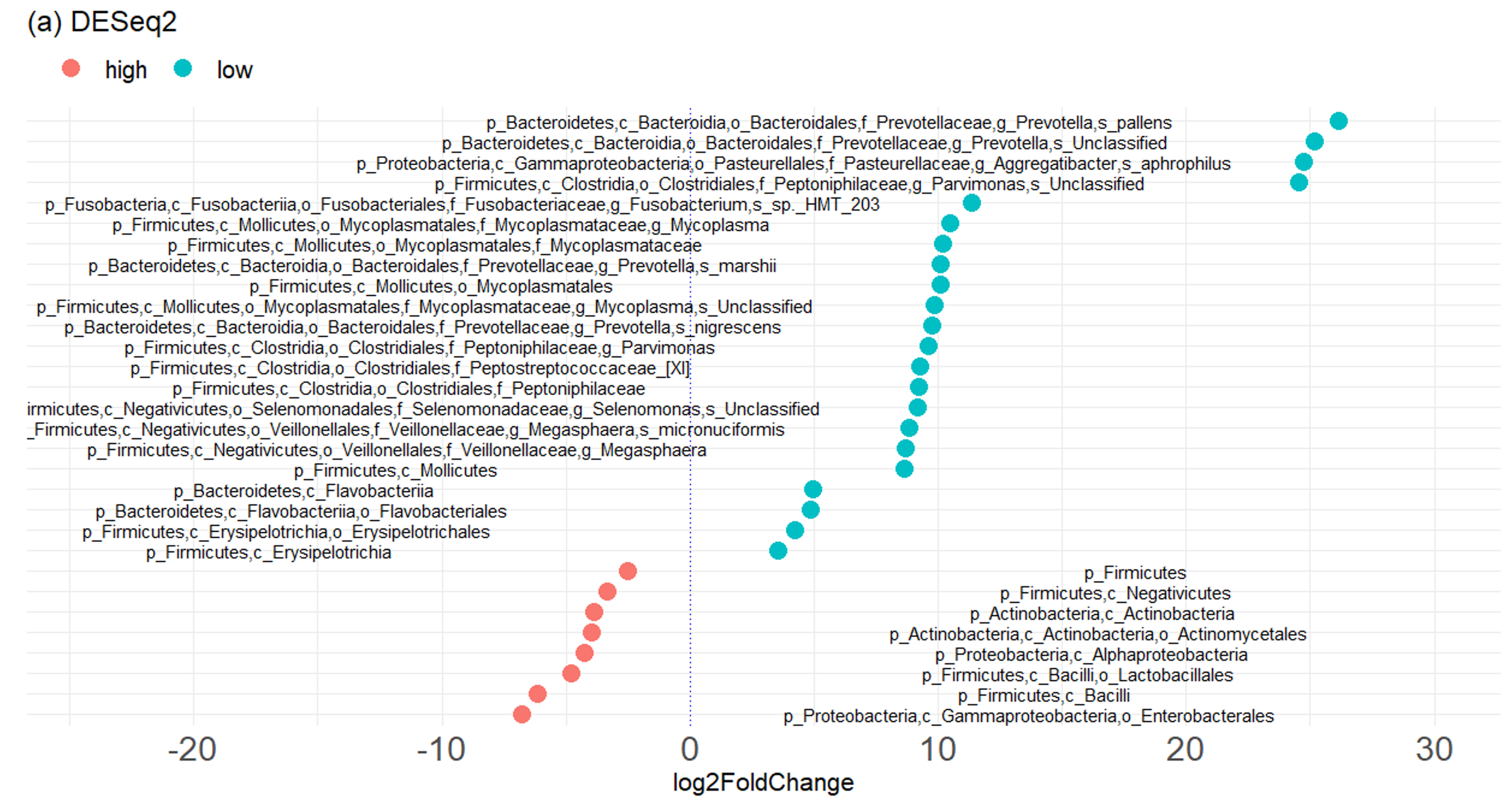

Supplement: Supplemental Information 1 — Result of DESeq2 analysis showing the log2 fold change of significantly different taxa [file peerj-10-14040-s001.png]

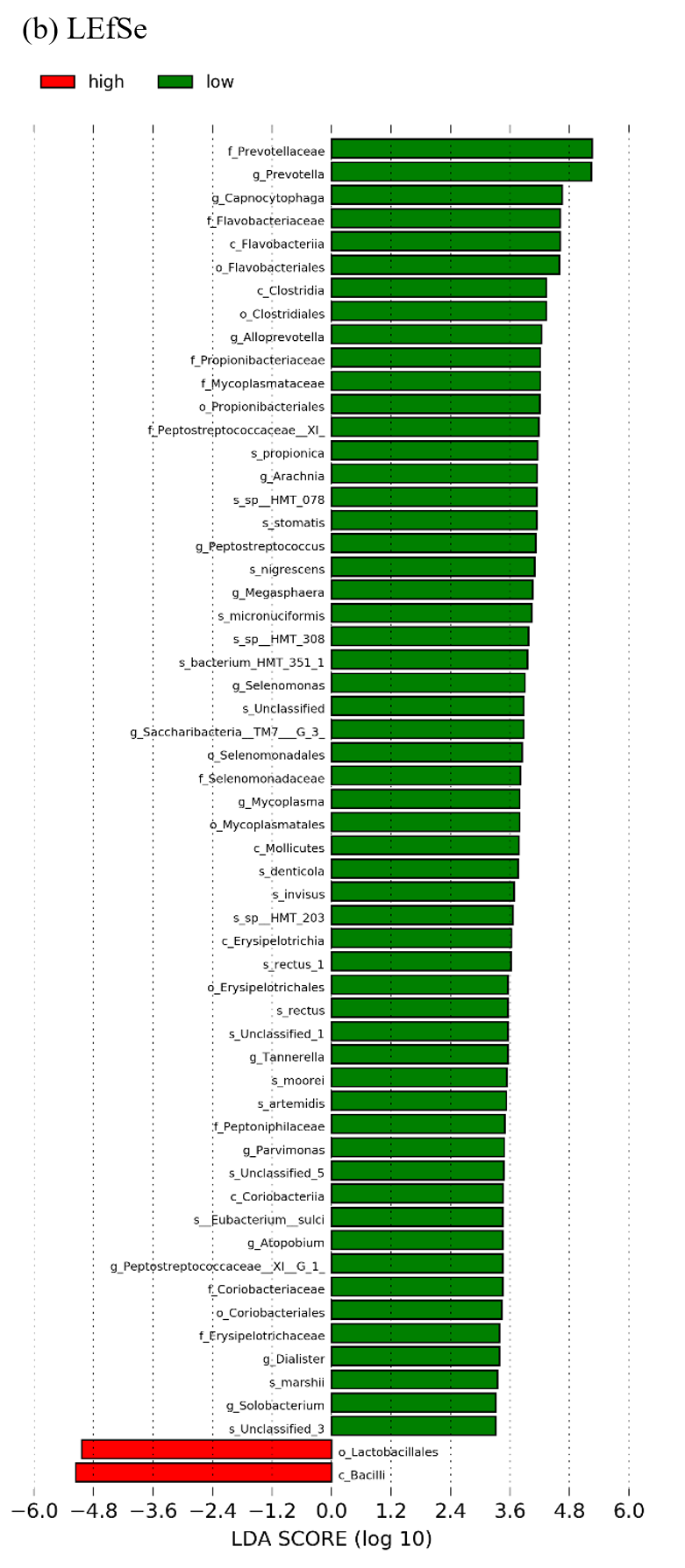

Supplement: Supplemental Information 2 — Result of LEfSe analysis showing the LDA score of significantly different microbial taxa. [file peerj-10-14040-s002.png]

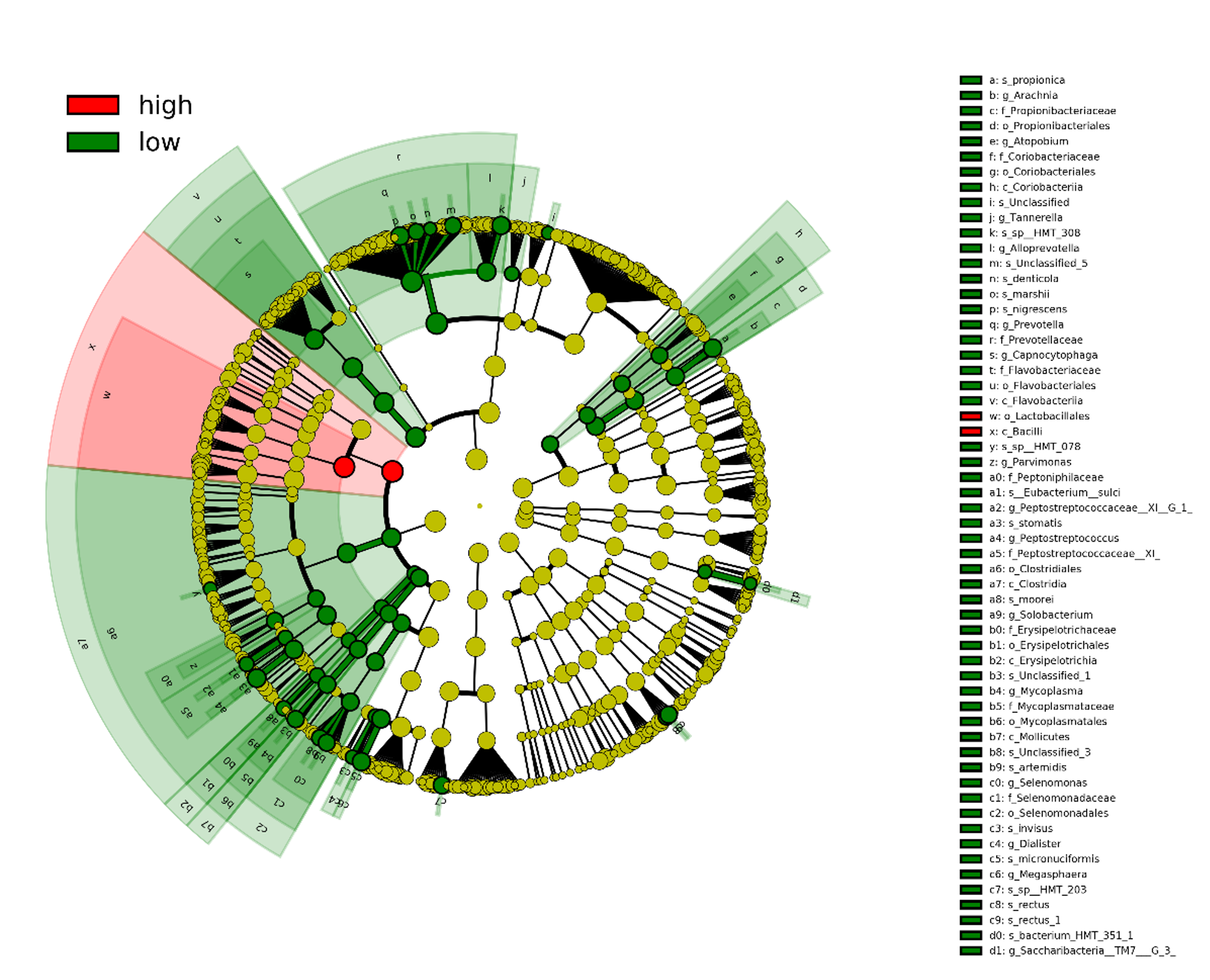

Supplement: Supplemental Information 3 — Differences are represented in the color of the most abundant class (red indicating high caries risk group, green indicating low caries risk group and yellow non-significant). Each circle’s diameter is proportional to the taxon’s abundance [file peerj-10-14040-s003.png]

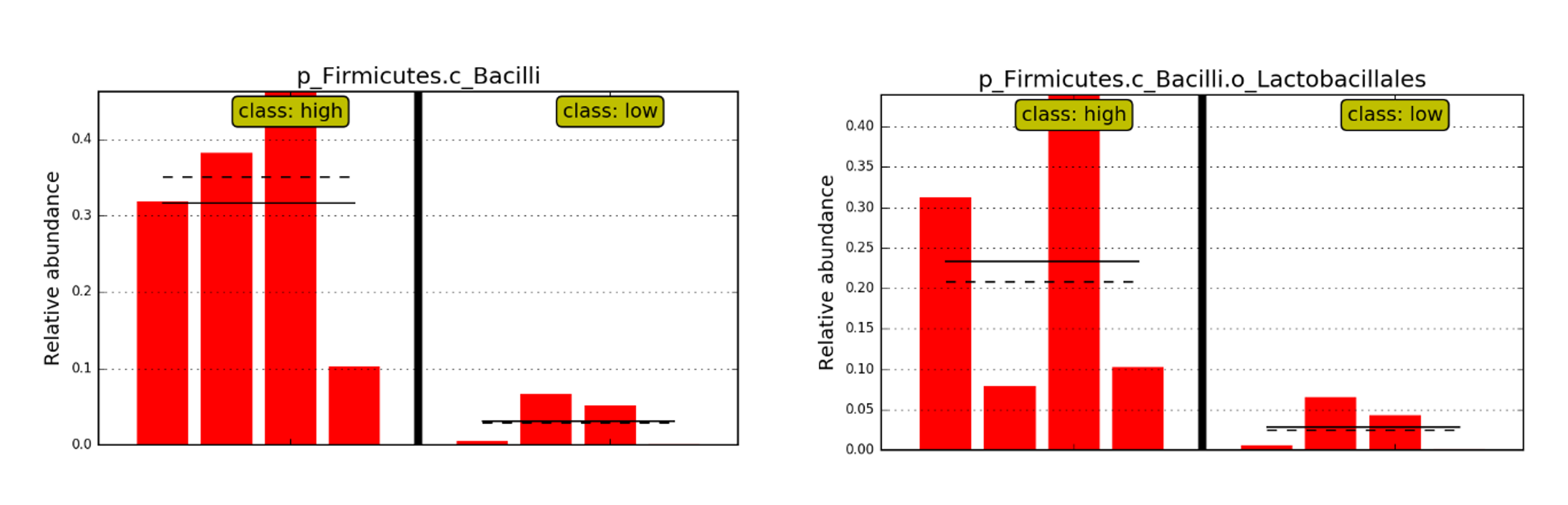

Supplement: Supplemental Information 4 [file peerj-10-14040-s004.png]
